# A Single Glycan at the 99-Loop of Human Kallikrein-related Peptidase 2 Regulates Activation and Enzymatic Activity*

**DOI:** 10.1074/jbc.M115.691097

**Published:** 2015-11-18

**Authors:** Shihui Guo, Wolfgang Skala, Viktor Magdolen, Peter Briza, Martin L. Biniossek, Oliver Schilling, Josef Kellermann, Hans Brandstetter, Peter Goettig

**Affiliations:** From the ‡ Department of Molecular Biology, University of Salzburg, 5020 Salzburg, Austria,; the § Klinische Forschergruppe der Frauenklinik, Klinikum Rechts der Isar der TU München, 81675 Munich, Germany,; the ¶ Institute of Molecular Medicine and Cell Research and; ‖ BIOSS Centre for Biological Signaling Studies, University of Freiburg, 79104 Freiburg, Germany,; the ** German Cancer Consortium (DKTK), 69120 Heidelberg, Germany,; the ‡‡ German Cancer Research Center (DKFZ), 69120 Heidelberg, Germany, and; the §§ Max-Planck-Institute for Biochemistry, 82152 Martinsried, Germany

**Keywords:** enzyme kinetics, kallikrein, N-linked glycosylation, prostate cancer, serine protease, substrate specificity, autolytic inactivation, zymogen activation

## Abstract

Human kallikrein-related peptidase 2 (KLK2) is a key serine protease in semen liquefaction and prostate cancer together with KLK3/prostate-specific antigen. In order to decipher the function of its potential *N*-glycosylation site, we produced pro-KLK2 in *Leishmania tarentolae* cells and compared it with its non-glycosylated counterpart from *Escherichia coli* expression. Mass spectrometry revealed that Asn-95 carries a core glycan, consisting of two GlcNAc and three hexoses. Autocatalytic activation was retarded in glyco-pro-KLK2, whereas the activated glyco-form exhibited an increased proteolytic resistance. The specificity patterns obtained by the PICS (proteomic identification of protease cleavage sites) method are similar for both KLK2 variants, with a major preference for P1-Arg. However, glycosylation changes the enzymatic activity of KLK2 in a drastically substrate-dependent manner. Although glyco-KLK2 has a considerably lower catalytic efficiency than glycan-free KLK2 toward peptidic substrates with P2-Phe, the situation was reverted toward protein substrates, such as glyco-pro-KLK2 itself. These findings can be rationalized by the glycan-carrying 99-loop that prefers to cover the active site like a lid. By contrast, the non-glycosylated 99-loop seems to favor a wide open conformation, which mostly increases the apparent affinity for the substrates (*i.e.* by a reduction of *K_m_*). Also, the cleavage pattern and kinetics in autolytic inactivation of both KLK2 variants can be explained by a shift of the target sites due to the presence of the glycan. These striking effects of glycosylation pave the way to a deeper understanding of kallikrein-related peptidase biology and pathology.

## Introduction

After early studies in the 1930s, when Frey, Kraut, and Werle characterized the first human kallikrein and derived a name from the Greek word for pancreas, καλλικρϵασ (reviewed in Refs. 1 and 2), the gene for this kallikrein (KLK1) was not discovered until 1985 (3, 4). During the late 1980s, two further genes with high structural similarity to *KLK1*, designated *KLK2* (glandular kallikrein) (5) and the most noted member *KLK3* (prostate-specific antigen) (6), were cloned and found to colocalize with *KLK1* to the same chromosomal region (19q13.4) (7). These three proteases share the unique extended “kallikrein loop” (8) that corresponds to the 99-loop of other (chymo)tryptic proteases. In KLK1–3, this loop partly covers the catalytic cleft (9) and seems to affect enzymatic specificity and turnover (8, 10). Moreover, variation within this loop is believed to contribute to functional diversity in kallikrein-related peptidases (KLKs)^3^ and their distinct physiological roles (11).

KLK2 displays 80% amino acid sequence identity with KLK3 (12). *In vivo*, KLK2 is synthesized as prepropeptide and modified through the secretory pathway. It is predominantly expressed in prostatic tissue (13, 14), and upon cleavage of the signal peptide, it is secreted as an inactive proenzyme, which is able to autoactivate to gain enzymatic activity (9, 15). KLK2^4^ is a tryptic serine protease (16) and is able to activate single chain urokinase-type plasminogen activator and pro-KLK3 (17). The later finding suggests a possible physiological role for KLK2 in the regulation of KLK3 activity. In addition, both KLK3 and KLK2 can digest the major gel-forming proteins in semen (*e.g.* semenogelin I (SEMG1) and II (SEMG2)) as well as fibronectin (16), resulting in the liquefaction of the seminal clot prior to impregnation. However, the majority of KLK2 protein in seminal plasma has been found in complex with protein C inhibitor (18). Its activity is also regulated by several protease inhibitors, such as α_2_-antiplasmin, α_1_-antichymotrypsin, antithrombin III, α_2_-macroglobulin, and plasminogen activator inhibitor-1 (19–22). Recently, KLK2 has been implicated in carcinogenesis and tumor metastasis of prostate cancer (23), whereas it is a useful serum marker of prostate cancer due to its association with prostatic proliferative disorders and tissue specificity (24, 25).

All KLKs are secreted proteases, and most of them will be glycosylated in the Golgi (26). KLK glycosylation might be required for proper folding, protection against proteolysis (autolysis), and regulation of enzymatic activity (26). KLK1 and -2 harbor one (KLK2) and two sequons (KLK1), respectively (*i.e.* typical amino acid triplets of the Asn-Xaa-Ser/Thr type for glycosylation) in their extended kallikrein/99-loop.

In KLK2, this single potential *N*-linked glycosylation site at Asn-95 is most probably glycosylated in the natural protease due to the apparent molecular mass of 31 kDa on SDS-PAGE (27). Protein glycosylation is an important post-translational modification in cell-cell recognition, ligand binding, antigen presentation, pathogen binding, and protein folding and stability (28–30). For example, changes in glycosylation patterns have been observed for KLK3 in prostate cancer (31) and for KLK6 ovarian cancer (32). However, the function of glycans in these proteases has not yet been fully understood. In the present study, we obtained glycosylated KLK2 from a eukaryotic expression system (LEXSY) and evaluated the role of *N*-glycosylation in KLK2 by comparison with the non-glycosylated form from *E. coli* or deglycosylated LEXSY-KLK2.

## Experimental Procedures

### 

#### 

##### Plasmid Construction, Leishmania Expression, and Purification of Human KLK2

Restriction enzymes and T4 ligase were obtained from Fermentas, (St. Leon-Rot, Germany), and Pfu Ultra II Fusion HS DNA polymerase was from Stratagene (La Jolla, CA). *E. coli* strains XL1 Blue and XL2 Blue (Stratagene) were used for subcloning expression constructs. All other chemicals, of the highest purity available, were either from Merck (Darmstadt, Germany), AppliChem (Darmstadt, Germany), or Sigma-Aldrich. The genes encoding mature KLK2 (Ile-16–Pro-245a) and the dead mutant KLK2S195A were cloned into the pQE-30 vector (Qiagen) with a His_6_ tag preceding the optimized enterokinase recognition sequence SGDR (9). Based on these templates, the encoding DNA fragments were amplified by PCR using a forward primer (CTAGTCTAGAGCATCACCATCACCATCACGGATCC) with an XbaI restriction site and a reverse primer (CTAGGCGGCCGCCTAGGGGTTGGCTGCGATGGTGTC) with a NotI restriction site (Eurofins MWG Operon, Martinsried, Germany). Subsequently, the PCR product was cloned in the pLEXSY-sat2 vector (Jena Bioscience) utilizing the XbaI and NotI restriction sites. The expression constructs carried an N-terminal signal sequence for secretory expression in the LEXSY medium.

The *Leishmania tarentolae* laboratory strain P10 (LEXSY, Jena Bioscience, Germany) was maintained at 26 °C as static suspension culture in 25-cm^2^ plastic cell culture flasks containing 10 ml of BHI medium (Jena Bioscience) containing 5 μg/ml hemin, 50 units/ml penicillin, and 50 μg/ml streptomycin, and was diluted 20-fold into fresh medium every 3–4 days. The expression constructs were linearized by SwaI digestion and electroporated into the LEXSY cells. Clones were selected following the manufacturer's instructions. Inoculation was done from a middle-late logarithmic phase growing preculture (*A*_600_ = 1.0). The expression of the secreted protein was evaluated by immunoblots of conditioned medium with an anti-His_5_ antibody. For large scale production of prostatic KLKs, cells were cultivated in 2-liter flasks filled with 500 ml of nutrient medium and shaken at 140 rpm at 26 °C in the dark for 48 h. The final *A*_600_ was about 3.0.

Media containing the secreted pro-KLK2 were harvested by centrifugation at 4,500 rpm for 30 min. The supernatant was incubated with nickel-nitrilotriacetic acid affinity resin (Qiagen Inc., Valencia, CA) at 4 °C for 4 h or overnight. Zymogen forms of KLK2 were eluted from the nickel-nitrilotriacetic acid resin with 50 mm imidazole and subsequently processed by C-terminally His-tagged enterokinase in a molar ratio of 1000:1 overnight at room temperature. To remove enterokinase and the KLK2 zymogen, the digested mixture was applied to benzamidine affinity chromatography and immobilized metal affinity chromatography (GE Healthcare, Uppsala, Sweden). Mature KLK2 bound to the benzamidine column and flowed through the metal affinity material. As a final step, size exclusion chromatography was performed with a Superose 6 10/300 GL column connected to an ÄKTA FPLC system (GE Healthcare) under 5 mm Tris-HCl, pH 8.0, 50 mm NaCl. Fractions that contained KLK2 were collected and concentrated.

##### Deglycosylation and Mass Spectrometry Determination

To remove *N*-linked oligosaccharides, 16 μg of purified pro-KLK2 from LEXSY cells were deglycosylated with 500 units of PNGase F (New England Biolabs, Frankfurt/Main, Germany) in 50 mm sodium phosphate, pH 7.5, at room temperature. The reaction was terminated with sample buffer, and proteins were detected on a 15% SDS-polyacrylamide gel.

For mass spectrometry, 50 μg of pro-KLK2 were incubated with 150 units of PNGase F for 24 h, leading to partial deglycosylation, and stored in a freezer before the test. The solution was digested by trypsin and the proteolytic fragments were sequenced by means of mass spectrometry with a Q-Exactive LC-MS/MS system (Thermo Scientific). Data analysis was performed with PEAKS 7 (Bioinformatics Solutions).

##### Enzymatic Assays and Proteomic Identification of Protease Cleavage Sites (PICS) Analysis

Bz-PFR-*p*NA, H-PFR-AMC, Chromozym X, and PPACK were obtained from Bachem (Weil am Rhein, Germany), whereas Ac-OFR-AMC was a product of California Peptide (Napa, California). KLK2 activity was determined in 50 mm Tris (pH 7.5), 100 mm NaCl, 10% (v/v) DMSO, 0.1% (w/v) bovine serum albumin (BSA) at 37 °C containing 70–500 nm KLK2, depending on the active fraction of the sample (see below). The maximum substrate concentration ranged from 0.7 to 10 mm, depending on the respective turnover and signal, respectively. The signal of the released *p*NA was measured photometrically at 405 nm, whereas that of released AMC was fluorimetrically recorded at excitation and emission wavelengths of 380 and 460 nm on an Infinite M200 microplate reader in triplicate or higher multiplicate samples (Tecan, Männedorf, Switzerland). Relative absorbance for the *p*NA standard (Sigma-Aldrich) and relative fluorescence for the AMC standard (Sigma) were measured at the same conditions and used in the calculations of the rate of product formation. The initial velocity of substrate turnover was fitted to the Michaelis-Menten equation by error-weighted non-linear regression analysis to obtain *k*_cat_ and *K_m_*. Substrate depletion was limited to <10%. The fraction of active KLK2 was determined by active site titration with the irreversible inhibitor PPACK by measuring the activity against H-PFR-AMC. *k*_cat_ values were calculated by dividing *V*_max_ through the active protease concentrations, which were 15.5% for KLK2_e_ and 45.3% for glyco-KLK2. Data fitting was performed with the ORIGIN software (OriginLab).

PICS analysis was done with samples of KLK2_e_ and LEXSY-derived glyco-KLK2 (33, 34). A variant of the original PICS method was used.^5^ Briefly, *E. coli* was grown in LB medium. Cells were lysed, and lysates were digested by either trypsin or GluC (34). The peptide digest was further purified by C18 solid phase extraction (Sep-Pak, Waters) according to the manufacturer's instructions. KLK2_e_ or glyco-KLK2 was incubated with the library at a 1:300 ratio for 3 h at 37 °C in 50 mm Tris (pH 7.5), 100 mm NaCl. The reaction was stopped by 1 μm PMSF. Protease-treated and control samples were isotopically labeled by triplex reductive dimethylation, as described elsewhere (35). Liquid chromatography-tandem mass spectrometry was performed on a Q-Exactive plus mass spectrometer coupled to an Easy nanoLC 1000 (both from Thermo Scientific) with a flow rate of 300 nl/min. Buffer A was 0.5% formic acid, and buffer B was 0.5% formic acid in acetonitrile (water and acetonitrile were at least HPLC gradient grade quality). A gradient of increasing organic proportion was used for peptide separation (5–40% acetonitrile in 80 min). The analytical column was an Acclaim PepMap column (2-μm particle size, 100-Å pore size, length 150 mm, inner diameter 50 μm) (Thermo Scientific). The mass spectrometer operated in data-dependent mode with a top 10 method at a mass range of 300–2000. For spectrum to sequence assignment, X! Tandem (version 2013.09.01) was used (36). The *E. coli* proteome database (strain K12, reference proteome) was used as described previously (37), consisting of 4304 protein entries and 8608 randomized sequences, derived from the original *E. coli* proteome entries. The decoy sequences were generated with the software DB toolkit (38). X! Tandem parameters included precursor mass error of ± 10 ppm (Q-Exactive), fragment ion mass tolerance of 20 ppm (Q-Exactive), semi-GluC specificity with up to one missed cleavage, and the following static residue modifications: cysteine carboxyamidomethylation (+57.02 Da) and lysine and N-terminal dimethylation (light formaldehyde, 28.03 Da; medium formaldehyde, 32.06 Da; heavy formaldehyde, 36.08 Da). X! Tandem results were further validated by PeptideProphet (39) at a confidence level of >95%. Peak areas for relative quantitation were calculated by XPRESS (40). Semispecific peptides that are increased >8-fold in the KLK2-treated samples were considered as KLK2 cleavage products. The corresponding prime or non-prime sequences were determined bioinformatically through database lookup. Web-PICS was used to generate a heat map style representation of protease specificity (41).

##### Autoproteolysis of KLK2

Active KLK2 was concentrated to 2.5 mg/ml, and the time-dependent activity loss at room temperature (∼23 °C) was monitored toward the most sensitive substrate H-PFR-AMC. The observed kinetic data fit well to the second-order reaction mechanism (*R*^2^ = 0.975). An equal amount of KLK2 autolysis samples was evaluated with 16% SDS-PAGE under reducing conditions and visualized by staining with Coomassie Brilliant Blue. The stained gels were scanned, and the percentage of autolysis KLK2 was evaluated based on the residual intact KLK2 protein using a pixel-based densitometer program, Un-Scan-It software (Silk Scientific).

##### Cleavage of Pro-KLK2 and Fibronectin

Degradation of pro-KLK2 S195A and fibronectin by prostatic KLK2 was visualized by SDS-PAGE and Coomassie Blue staining. For fibronectin degradation, 50 μg of purified human serum fibronectin (Innovative Research, Novi, MI) were incubated with 0.5 μg of KLK2 from *E. coli* or LEXSY cells for different periods of time (0, 1, 2, 4, and 24 h) at 37 °C in 50 mm Tris-HCl, pH 8.0, containing 0.1% Nonidet P-40, 150 mm NaCl. As a control, corresponding amounts of fibronectin or protease alone were monitored at the identical condition. Degradation of pro-KLK2 S195A was carried out by incubating 25 μg of pro-KLK2 S195A with 5 μg of active KLK2 (*E. coli* or LEXSY) or 48 ng of enterokinase at 37 °C, for 0, 1, 2, 4, and 24 h. The cleavage was terminated by mixing with SDS loading buffer and boiling for 5 min immediately after a given time point.

## Results

### 

#### 

##### Recombinant Expression, Purification, and Characterization

**FIGURE 1.**
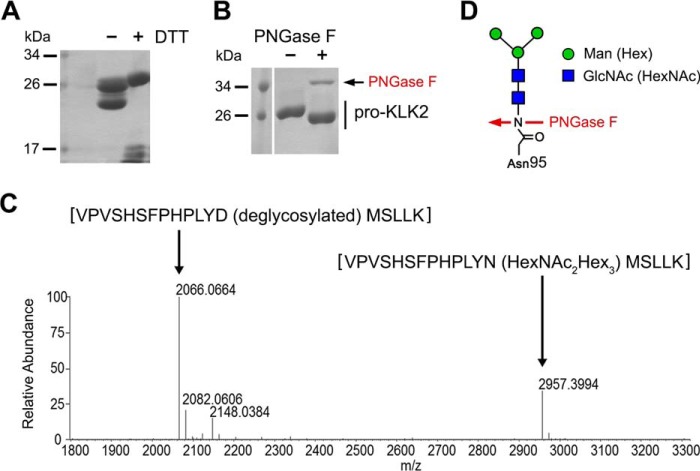
**Analysis of KLK2 glycosylation.** *A*, enterokinase-activated KLK2 from *Leishmania* expression (LEXSY) was analyzed under non-reducing and reducing conditions. Clipped products migrated as a single band under non-reducing conditions. Intact and clipped forms, which form fragments smaller than 17 kDa under reducing conditions (+*DTT*), were not separable and appeared as a single peak in gel filtration. *B*, removal of *N*-glycans from LEXSY-KLK2 with PNGase F (36 kDa) resulted in a shift of the apparent molecular mass by about 1 kDa. *C*, trypsin-digest mass spectrometry confirmed partially deglycosylated LEXSY-KLK2 by PNGase F under non-denaturing conditions. The deconvoluted mass spectrum is shown, and the indicated masses refer to uncharged molecules. The peak at 2957 was identified by sequencing and subsequent modification search as VPVSHSFPHPLYN-(Hex_3_HexNAc_2_)-MSLLK. The signal at 2066 represents the corresponding deglycosylated peptide (VPVSHSFPHPLYD-(deglycosylated)-MSLLK), with the expected core glycan mass of 891 Da. *D*, *N*-linked core glycans at Asn-95 of LEXSY KLK2, consisting most likely of two *N*-acetylglucosamine and three mannose units. PNGase F cleaves the *N*-glycan and generates an Asp-95.

In order to produce mature KLK2 (Ile-16–Pro-245a), a short artificial propeptide encoding a His tag followed by an optimized cleavage site for enterokinase, His_6_-(Gly-Ser)-Ser-Gly-Asp-Arg, was added to the N terminus. Pro-KLK2 was secreted by *Leishmania* cells with an average protein yield of about 0.9 mg/liter of culture, and its identity was confirmed by mass spectrometry. After immobilized metal affinity chromatography, it appeared as a single band on an SDS-polyacrylamide gel. The propeptide was removed with enterokinase, generating mature KLK2, which was subjected to gel filtration on a Superdex 75 column. The protein eluted as a single peak with an apparent molecular mass of 27 kDa corresponding to the monomer. Unfortunately, mature KLK2 could not be separated from enterokinase clipping products because the fragments were held together by disulfide bonds and exhibited similar properties as mature, intact KLK2 on ion exchange, benzamidine affinity chromatography, and size exclusion chromatography columns (Fig. 1*A*). Edman degradation confirmed the correct N-terminal sequence Ile-Val-Gly-Gly-Trp-Glu-*X*-Glu-Lys (where *X* corresponds to Cys in the KLK2 sequence). Furthermore, mature KLK2 was active toward the chromogenic substrate Bz-PFR-*p*NA.

Expression of pro-KLK2 in *Leishmania* cells lead to a defined band of ∼27 kDa on SDS-polyacrylamide gels, which shifted upon PNGase F treatment to 26 kDa, corresponding to a loss of a short *N*-glycan from the KLK2 (Fig. 1*B*). The glycosylation was further confirmed by glycan staining (periodic acid-Schiff staining) and mass spectrometry, the latter indicating attachment of two GlcNAc units and three hexoses at Asn-95, (Fig. 1*C*), which are most likely the mannose units of a eukaryotic core glycan linked to the only potential *N*-glycosylation site of KLK2 (Fig. 1*D*). The glycan cannot be cleaved by EndoH, which removes only *N*-linked sugars containing more than three mannose moieties (42). This short glycan increases the molecular mass from 26.18 to 27.08 kDa, in agreement with the observed bands on SDS gels.

##### Activation of Pro-KLK2

**FIGURE 2.**
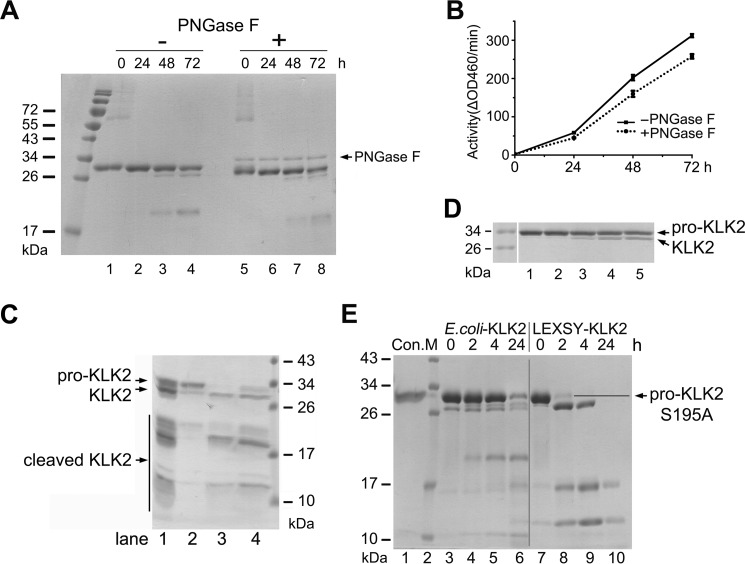
**Autoactivation and activation of pro-KLK2.** *A*, time-dependent autoactivation of both glycosylated (*lanes 1–4*) and enzymatically deglycosylated pro-KLK2 (*lanes 5–8*) is monitored on an SDS-PAGE. Note the same shift of about 1 kDa as in Fig. 1*B. B*, activity assay of autoactivated KLK2 with the small peptide substrate H-PFR-AMC by glycosylated (*black line*) and enzymatically deglycosylated KLK2 (*dotted line*) at varying times (0, 24, 48, and 72 h), matching those in *A. C*, concentration-dependent autoactivation of pro-KLK2-S195A. The initial pro-KLK2 samples at concentrations of 250 (*lane 1*), 600 (*lane 2*), 800 (*lane 3*), and 900 (*lane 4*) μg/ml were kept at room temperature for 1 day and subsequently at 4 °C for 4 days, respectively. *D*, activation of pro-KLK2. Shown is incubation of 1 μg of pro-KLK2-S195A from LEXSY cells (*lane 1*) with 1% KLK2_e_ from *E. coli* (*lane 2*), 1% glyco-KLK2 from LEXSY cells (*lane 3*), or 0.05% (*lane 4*) or 0.1% enterokinase (*lane 5*) overnight (14 h) at room temperature. *E*, pro-KLK2-S195A (*lane 1*) was digested by 20% KLK2_e_ (*lanes 3–6*) or 20% glyco-KLK2 (*lanes 7–10*) for a time-dependent course. Samples were taken at 0, 2, 4, and 24 h, as indicated *above* the *lanes*, and then checked by Coomassie Blue-stained SDS-PAGE. The *vertical gray line* was inserted to indicate samples with different treatment. KLK2_e_ shows a different cleavage pattern (*e.g.* by generating a 20-kDa fragment, which originates from the clipping at Arg-70↓His-71).

The same artificial propeptide as described above, comprising a His_6_ tag and an optimized recognition sequence for enterokinase activation, was employed in the production of pro-KLK2 in *E. coli* (referred to as pro-KLK2_e_ hereafter). After *in vitro* folding, KLK2_e_ turned out to be fully maturated, indicating a spontaneous autoactivation. By contrast, during LEXSY cell expression, the soluble pro-KLK2 was secreted into the medium and had initially no enzymatic activity toward the Bz-PFR-*p*NA substrate and underwent only slow autoactivation. This activation was corroborated by an accumulation of the KLK2 band on SDS-PAGE (Fig. 2*A*, *lanes 2–4*) and a corresponding slow increase of fluorogenic activity (Fig. 2*B*, *black line*). The 20 kDa band, which occurs after 48 h, is part of a KLK molecule that was clipped at the Arg-70–His-71 bond but is held together by two disulfides under oxidizing conditions (12). However, the autoactivation is strongly time- and concentration-dependent: increasing the pro-KLK2 concentration in the range of 250–900 μg/ml did not enhance but rather decreased generation of mature KLK2 after 24 h (Fig. 2*C*). This apparent paradox can be explained by the second-order reaction of the in *trans* autocleavage of KLK2, which results in accelerated inactivation with higher protein concentration (Fig. 2*C*). We further investigated whether glycosylation influences the activation and autoactivation process of pro-KLK2 (Fig. 2*D*). Autoactivation of glycosylated KLK2 and enzymatically deglycosylated KLK2 was qualitatively similar and slow as judged by SDS-PAGE (Fig. 2*A*), which correlates well with the fluorogenic activity of glyco- and deglyco-KLK2 samples (Fig. 2*B*).

To reduce the complexity of the autoactivation reaction, we produced the dead mutant pro-KLK2-S195A in LEXSY cells to abolish the positive feedback of the activated product. We added 20% KLK2 either from *E. coli* or LEXSY to “activate” the pro-KLK2-S195A dead mutant (*i.e.* to cleave the propeptide and liberate the N-terminal Ile-16). Intriguingly, glycosylated KLK2 turned out to be a considerably better activator than the non-glycosylated enzyme (Fig. 2*E*), albeit still ∼200 times slower than enterokinase (Fig. 2*D*). Nearly all pro-KLK2-S195A molecules had been turned over by glyco-KLK2 to the mature species within 2 h, whereas only about 10% of pro-KLK2 had been converted in 4 h by KLK2_e_ in a non-productive manner. KLK2_e_ preferentially produces an inactive 20-kDa fragment (43) that is further degraded (Fig. 2*E*, *lanes 4–6*). By contrast, the mature KLK2 form (26 kDa) was further processed by glyco-KLK2 to different fragments of ∼17 and 11 kDa (Fig. 2, *C* and *E*).

##### Autolysis of KLK2

**FIGURE 3.**
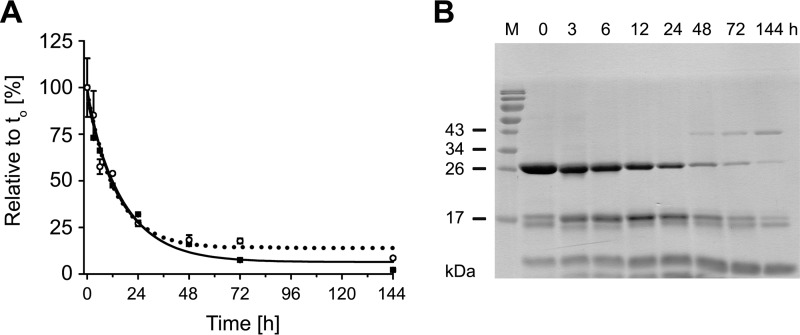
**Time-dependent autoinactivation of KLK2.** *A*, the decrease of intact glyco-KLK2 (*black squares* and *line*) correlates well with the loss of protease activity (*open circles* with *dotted line*). The loss of mature protein and activity follows a second-order reaction mechanism. *B*, time-dependent KLK2 autolysis was monitored by reducing SDS-PAGE.

As already described, autoactivation is followed by autoproteolysis (Fig. 2*E*). In non-glycosylated KLK2 from *E. coli*, autolysis was paralleled by loss of enzymatic activity (9). To identify the potential effect of glycosylation on KLK2, time-dependent loss of KLK2 enzymatic activity was monitored over 144 h. Like *E. coli* KLK2 (KLK2_e_), active KLK2 did undergo autolysis with a loss of activity (Fig. 3*A*). Fitting of the inactivation data to the second-order rate law, −d[KLK2]/d*t* = *k*·[KLK2]^2^ and [KLK2]*_t_*_= 0_ = 100%, resulted in the rate constant *k* = 0.017 ± 0.001 day^−1^. Interestingly, the inactivation rate constant is lower than the similarly determined inactivation rate constant of KLK2_e_ (0.028 day^−1^) (9). Apparently, glycosylation slows down the autolysis, in agreement with a stabilizing effect of glycosylation with respect to proteolysis (44).

The major autolysis band appeared with a molecular mass of about 17 kDa (Fig. 3*B*). Sequencing of the 17 kDa band identified the five N-terminal residues of the full-length mature enzyme (starting with Ile-16), suggesting a cleavage in the 148-loop. Previously, a cleavage site between residues Arg-153 and Ser-154 has been reported for natural KLK2 from seminal plasma and insect KLK2 (18, 45). This cleavage generates two fragments: one with a molecular mass of 17.2 kDa, which consists of residues Ile-16 to Arg-153 carrying the short glycan or oligosaccharide, and one of 9.9 kDa, corresponding to the fragment Ser-154 to Pro-254a. Two additional bands were observed with KLK2_e_ (Fig. 2*E*, *lanes 3–6*), which might be attributed to the established cleavage between Arg-95e and His-95f, resulting in a 10.2 kDa band, including an oligosaccharide attached to the Ile-16 to Arg-95e fragment, whereas the 16.9 kDa band corresponds to His-95f to Pro-254a, as confirmed by N-terminal sequencing for KLK2_e_ (9, 12).

##### Influence of Glycosylation on KLK2 Protease Specificity and Enzyme Kinetics

**FIGURE 4.**
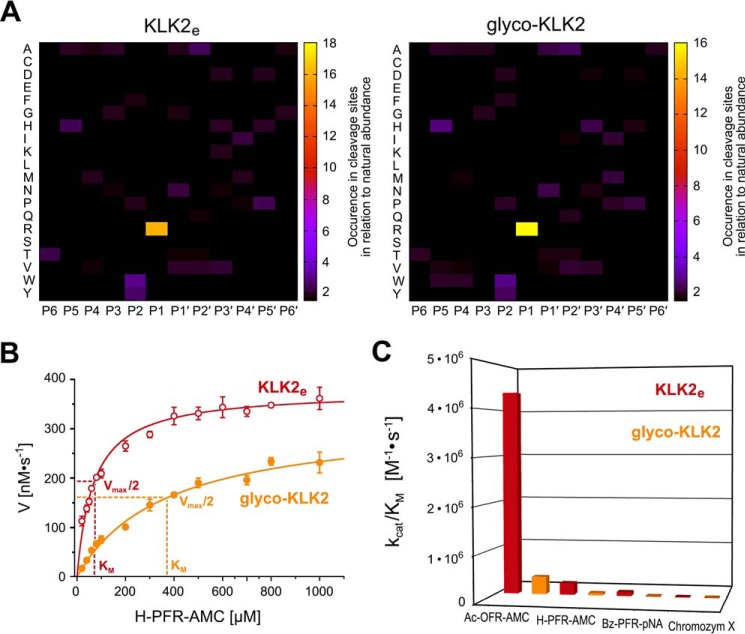
**Effects of glycosylation on KLK2 activity.** *A*, PICS of KLK2_e_ (*left*) and glyco-KLK2 (*right*) covering peptide substrates from residue P6 to P6′. Increased occurrence of an amino acid residue in cleavage sites of a random peptide library indicates the corresponding preference of the peptidase specificity sites S6–S6′. Both patterns are similar; P1-Arg dominates the specificity, whereas aromatic P2 residues and hydrophobic P4 residues are slightly favored. In P1′ and P2′, small or polar residues and small to medium hydrophobic residues are preferred, respectively. *B*, Michaelis-Menten plot of H-PFR-AMC turnover by KLK2_e_ (*open red circles*) and glyco-KLK2 (*filled orange circles*). The velocity was normalized for 150 nm enzyme concentration, according to active site titrations. It is noteworthy that the *K_m_* of glyco-KLK2 is 5 times higher than that of KLK2_e_, despite a comparable *V*_max_ and *k*_cat_ of 17.0 and 13.6 s^−1^, respectively. *C*, catalytic efficiency depicted as *columns* for KLK2_e_ (*red*) and glyco-KLK2 (*orange*) for fluorogenic and chromogenic substrates (Table 1). Chromozym X is Mco-d-Nle-Gly-Arg-*p*NA. Whereas the *k*_cat_ varies considerably, the *K_m_* for all substrates is consistently about 5 times higher with glyco-KLK2 than with KLK2_e_.

Human KLK2 exhibits a predominantly trypsin-like activity with a strong preference for Arg residues in the P1 position, according to the Schechter and Berger nomenclature for peptidic substrate residues P and protease specificity subsites S (9, 46, 47). Mass spectrometry-based profiling of protease specificity with proteome-derived peptide libraries is a powerful method to simultaneously identify prime and non-prime specificity determinants (*i.e.* on the N-terminal and the C-terminal side of the scissile bond, respectively) (48). Here, we used a recently developed variant of the original PICS approach, which enables profiling of lysine specificity.^5^ The peptide library was prepared from *E. coli* using GluC digestion, thus enabling identification of specificity for basic residues. Specificity profiling of KLK2_e_ and glyco-KLK2 with this library from P6 to P6′ showed an overall high similarity, with P1-Arg as major specificity determinant, with 88–89% cleavage sites (Fig. 4*A*). For both KLK2_e_ and glyco-KLK2, ∼8% of the cleavage sites feature a P1-Lys, thus defining P1 specificity as Arg ≫ Lys for both KLK2 variants. In P2, a preference for aromatic residues is observed because Phe, Trp, and Tyr together account for about 17% of both KLK2_e_ and glyco-KLK2 cleavage sites. If this number is corrected for the natural abundance of aromatic amino acids, their percentage rises to about 30%. In addition, Asp seems to be a moderately preferred residue in P2 (8%). P3 is overall unspecific and, in particular, disfavors Pro, which is not present in any cleavage product. Also, P4 is not very specific, although the hydrophobic, aliphatic residues Ala, Val, Ile, Leu, and Met are found in 45% of cleavages, which decreases to 34% by the natural abundance correction. Otherwise, the covered prime-side specificity pockets appear to be similarly unspecific. In P1′, the small, polar residues Ser, Thr, and Asn account for 27% (KLK2_e_) and 28% (glyco-KLK2) of cleavage sites, respectively, whereas Ala and Val contribute an additional 28% in both cases. A tendency toward small, more hydrophobic residues is observed in the P2′ position, with Ala, Val, Ile, and Thr seen in 51% of all cleavage products. Although some residues, such as Asp, His, and Val or Glue, Ile, and Pro, respectively, occur more often in the P3′ and P4′ positions, no distinct specificity can be assigned there.

The peptidic substrate turnover was determined using the small fluorogenic and chromogenic substrates Ac-Orn-Phe-Arg-AMC (where Orn represents ornithine), H-Pro-Phe-Arg-AMC, Bz-Pro-Phe-Arg-*p*NA, and Mco-d-Nle-Gly-Arg-*p*NA (where Nle represents norleucine; also known as Chromozym X), respectively (Fig. 4*B*). The recently published data of KLK2_e_ for H-PFR-AMC and Bz-PFR-*p*NA were also included in Fig. 4*C* and Table 1 for comparison (9). For all P1-Phe substrates, the *K_m_* of glyco-KLK2 was consistently ∼5 times higher than that of KLK2_e_, whereas it was even 12 times higher for Chromozym X, representing a general lower affinity for small peptidic substrates, lacking amino acids in the prime-side region. Furthermore, the *k*_cat_ of glyco-KLK2 was about 2-fold lower compared with the *k*_cat_ of KLK2_e_ for Ac-OFR-AMC and Bz-PFR-*p*NA, whereas it was nearly equal for H-PFR-AMC but about 10 times higher for the turnover of Chromozym X (Table 1). The overall catalytic efficiency (*k*_cat_/*K_m_*) of KLK2_e_ for the best substrate, Ac-OFR-AMC, was 4,368,890 m^−1^ s^−1^ (*i.e.* more than 13,000 times higher than for the worst, Chromzym X, the only one with Gly instead of Phe in the P2 position). For the three P2-Phe substrates, the catalytic efficiency of glyco-KLK2 ranged from 7 to 15% of that of KLK2_e_, whereas it was nearly double the value of KLK2_e_ for Chromozym X.

**TABLE 1:** **Kinetic parameters of KLK2 toward synthetic substrates** For the Michaelis constants *K_m_* and the turnover number *k*_cat_, the weighted S.E. values of the non-linear regression fitting were used, whereas the error of the catalytic efficiency was calculated according to the formula given by Fenner (76).

KLK2 source*^a^*	Substrate	*K_m_*	*k*_cat_	*k*_cat_/*K_m_*
		μ*m*	*s*^−*1*^	*m*^−*1*^ *s*^−*1*^
*E. coli*	Ac-OFR-AMC*^b^*	45 ± 2	196.60 ± 5.04	4,368,890 ± 224,160
LEXSY	Ac-OFR-AMC	224 ± 11	96.05 ± 3.97	308,260 ± 27,520
*E. coli*	H-PFR-AMC*^c^*	69 ± 3	17.03 ± 0.14	246,810 ± 10,920
LEXSY	H-PFR-AMC	378 ± 27	13.57 ± 0.54	35,890 ± 2940
*E. coli*	Bz-PFR-pNA*^c^*	75 ± 2	5.77 ± 0.09	76,930 ± 2380
LEXSY	Bz-PFR-pNA	448 ± 44	2.59 ± 0.17	5780 ± 680
*E. coli*	Mco-d-Nle-GR-*p*NA*^d^*	302 ± 14	0.10 ± 0.01	330 ± 40
LEXSY	Mco-d-Nle-GR-*p*NA	3567 ± 317	1.95 ± 0.10	550 ± 60

*^a^* Expression system.

*^b^* Acetyl-Orn-Phe-Arg-AMC contains the non-proteinogenic amino acid ornithine in P3 position, which is a homolog of Lys, being shorter by one methylene group.

*^c^* The values for KLK2_e_ with H-PFR-AMC and Bz-PFR-*p*NA correspond to the data of Skala *et al.* (9) but were corrected according to a new active site titration with PPACK, in which KLK2_e_ was 15.5% active, whereas the LEXSY protein showed 45.3% activity.

*^d^* Mco-d-Nle-Gly-Arg-*p*NA, also known as Chromozym X, has the non-natural d-norleucine in the P3 position, whereas its side chain occupies the S4 rather than the S3 subsite.

##### KLK2 Glycosylation Alters the Cleavage Pattern of Protein Substrates

As observed in the activation studies using the dead mutant glyco-pro-KLK2 S195A (Fig. 2), *E. coli*- and *Leishmania*-derived KLK2 exhibited a marked difference in the cleavage pattern of this mutant. Apparently, glycosylation of the enzyme strongly affects the preference of substrate recognition sites. Non-glycosylated KLK2_e_ hardly converted pro-KLK2 S195A into mature KLK2, in contrast to the processing by glyco-KLK2 (Fig. 2*C*). Also, further degradation products differed distinctly. These observations suggest that the enzyme glycosylation serves as a checkpoint for KLK2 autoactivation; only properly glycosylated KLK2 can feed back in the activation of glycosylated pro-KLK2. However, it is interesting to note that non-glycosylated KLK2_e_ is able to produce quantitative amounts of mature KLK2_e_ as well, requiring more time (9).

**FIGURE 5.**
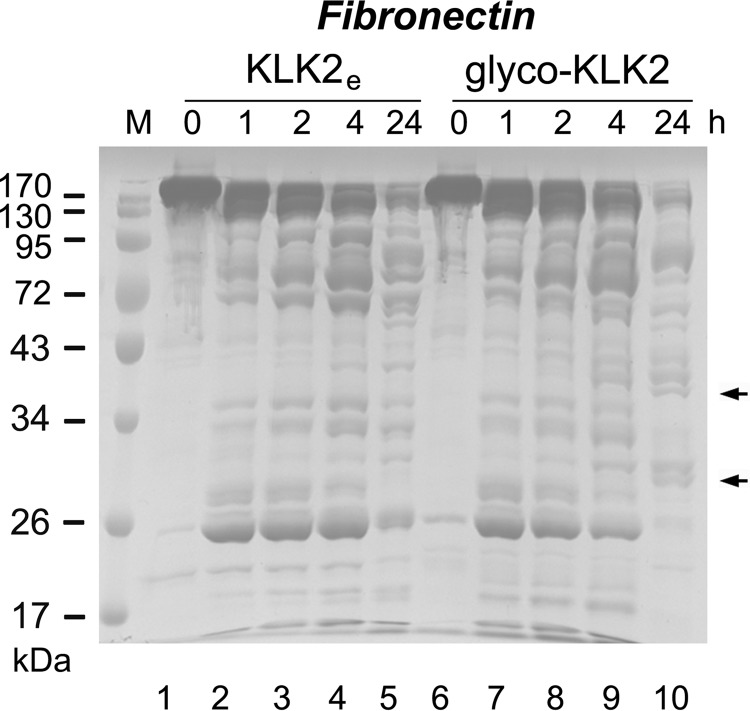
**Comparative degradation rates and fragmentation patterns of human fibronectin in the presence of KLK2_e_ (*lanes 2–6*) glyco-KLK2 (*lanes 7–11*) as determined by SDS-PAGE.** Aliquots were taken at representative intervals and analyzed by SDS-PAGE followed by Coomassie Blue staining. Fibronectin alone is stable at 37 °C for at least 24 h. Overall, the breakdown by glyco-KLK2 appears more efficient and results in some unique bands after 24-h incubation, as indicated by *arrows*.

The different processing patterns of glyco-KLK2 and KLK2_e_ on pro-KLK2-S195A reflect differences in substrate turnover and specificity that are not based on simple recognition of amino acids sequences. To investigate whether such differences in protein substrate recognition occur more generally, we investigated the digestion of the natural, physiologically relevant substrate fibronectin, which is present in seminal plasma (22). The incubation of fibronectin with either *E. coli*- or LEXSY-derived KLK2 produced a large number of peptides (Fig. 5). In the first 4 h, the digestion patterns of fibronectin by both KLK2 forms look nearly identical, and they still look similar after 16 h. Subsequently, glyco-KLK2 digested the fibronectin fragments somewhat more rapidly, resulting in additional bands after 24 h, which can be found in the KLK2_e_ sample after a more extended incubation up to 3 days. Thus, the enzymatic activity of glyco-KLK2 is substantially altered when it comes to the turnover of larger protein substrates with distinct tertiary structure and recognition sites that do not belong to small synthetic substrates. Remarkably, the short single glycan linked at Asn-95 makes KLK2 a more efficient and more specific protease for natural substrates, such as pro-KLK2 and fibronectin.

## Discussion

Glycosylation is a key feature of nearly all human kallikreins, although they vary considerably with respect to location and glycan composition (26). We produced *N*-glycosylated KLK2 in *Leishmania* cells (LEXSY), which generate short glycans that correspond to the core of the more complex mammalian glycans (49). In our hands, KLK2 could not be obtained from mammalian expression in HEK293 T/S cells. Glycoproteins secreted from *Leishmania* cells are smaller (49) and very homogeneous (Fig. 1) when compared with glycoproteins from natural sources (18, 27, 50) or insect expression (51). Mass spectrometry confirmed the presence of a eukaryotic core glycan linked to Asn-95, which typically consists of two GlcNAc and three mannose units (Fig. 1*C*).

Previously, we obtained pure and active KLK2_e_ from *E. coli* expression (9). A comparative proteomic specificity profiling (PICS) of both KLK2 variants resulted in nearly identical profiles for the P6 to P6′ positions (Fig. 4*A*). The high preference for P1-Arg over Lys with 89% *versus* 8% cleavages resembles the result of a phage display study with 98% *versus* 2%, in contrast to the positional scanning measurements, where Lys reached 45% of Arg at 100% (9, 47). Whereas the P2 position seems unspecific in the phage display, it appears specific for aromatic residues, although not as pronounced as in the positional scanning. All three approaches agree on the unspecific nature of the S3 subsite and on the acceptance of hydrophobic, aliphatic P4 residues. The proteomic identification of protease cleavage sites and phage display find small and polar residues in P1′, whereas a tendency to hydrophobic P2′ residues is weaker in the phage display. Both methods confirm the unspecific character of position P3′ and beyond.

The enzyme kinetics of four small P1-Arg substrates showed that glyco-KLK2 always has a significantly decreased binding affinity, represented by an at least 5-fold higher *K_m_*, whereas variations of the turnover rate (*k*_cat_) were not correlated (Table 1). Ac-OFR-AMC exhibits the highest catalytic efficiency, probably because the otherwise non-proteinogenic amino acid ornithine forms ideal backbone hydrogen bonds to both the Gly-216 amide NH and carbonyl oxygen, in contrast to Bz-PFR-*p*NA that can bind only with the Pro carbonyl oxygen to the Gly-216 NH. According to PICS, P3-Pro is a distinctly disfavored residue. However, the N-terminal P3-Pro in H-PFR-AMC can form both hydrogen bonds to Gly-216, which may explain its higher catalytic efficiency compared with Bz-PFR-*p*NA (Table 1). Otherwise, KLK2 might prefer AMC over *p*NA in the prime side, although both substrate types can have similar kinetic parameters as for trypsin (52).

**FIGURE 6.**
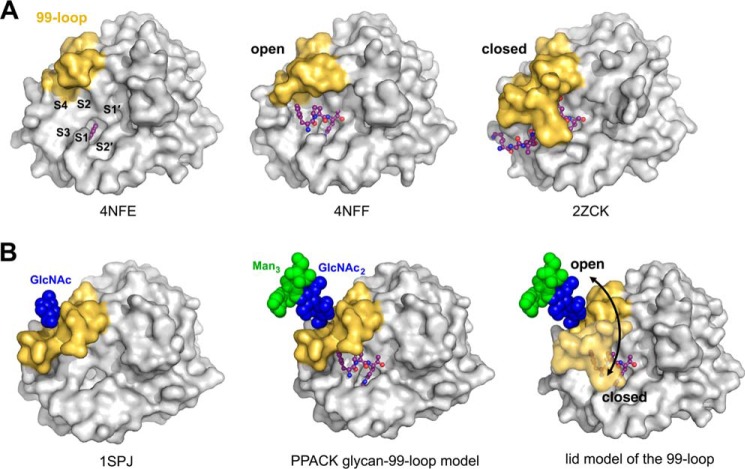
**Model of the 99-loop lid mechanism and possible regulation of KLK2 activity by glycosylation.** *A*, the *left panel* shows the crystal structure of KLK2_e_ (Protein Data Bank code 4NFE) with the small inhibitor benzamidine as a *ball-and-stick model* located in the S1 pocket, which is labeled like the other pockets from S4 to S2′, whereas the molecular surface of the wide open 99-loop is depicted in *yellow*. In the related KLK2_e_ PPACK complex (Protein Data Bank code 4NFF), P1-Arg, P2-Pro, and d-Phe occupy the non-prime region, from the S1 to the S4 subsite, respectively. It exhibits an open 99-loop, which can hardly interfere with substrate binding. In the *right panel*, the structure of a human KLK3 acyl intermediate complex (Protein Data Bank code 2ZCK) represents a fully closed, non-glycosylated 99-loop, which is homologous to the KLK2_e_ counterpart. Parts of the 99-loop interact tightly with the P2–P4 residues, contributing considerably to substrate specificity. *B*, in the *left panel*, the apo-KLK1 structure (Protein Data Bank code 1SPJ) contains a 99-loop with a glycosylated Asn-95, displaying a half-open conformation, most likely due to the presence of the stabilizing glycan, of which only a GlcNAc was visible. This structure is the basis of the PPACK glycan-99-loop model (*middle*), extended by one GlcNAc and three mannose units of a core glycan. PPACK and *p*NA or AMC substrate binding would be hampered by the rather occluding 99-loop conformation. The combined model of glycosylated 99-loop with a bound substrate in the S2–S4 region (*right*) suggests that the active site is less accessible in the closed state but also more stable during the following catalytic steps. A favored closed state will result in a lower *k*_on_ rate and higher *K_m_*, consistent with the observed enzyme kinetics of glyco-KLK2 and KLK2_e_.

Since both specificity profiles of the proteomic identification of protease cleavage sites show no significant differences after cut-off of the background (Fig. 4*A*), the explanation for the effect of the Asn-95 glycosylation is most likely an altered active site conformation near the specificity pockets S2–S4. In the proteomic approach, KLK2_e_ exhibited more unspecific background cleavages than glyco-KLK2, which suggests an increased adaptability of the active site to substrates. Most likely, the more flexible 99-loop of KLK2_e_ favors a wide open conformation that allows small substrates faster binding to the active site. This process could be described by a higher *k*_on_ rate, resulting in a decreased *K_m_*, which equals (*k*_off_ + *k*_cat_)/*k*_on_. In line with this proposal, the *K_m_* for all investigated substrates with KLK2_e_ was always lower than 20% of the corresponding *K_m_* of glyco-KLK2, independent of the catalytic efficiency (Table 1). Two KLK2_e_ structures (Protein Data Bank codes 4NFE and 4NFF) feature such an open active site and a disordered, flexible 99-loop (Fig. 6*A*). By contrast, apo-KLK1 (Protein Data Bank code 1SPJ) is equally glycosylated at Asn-95 but has a more rigid and half-closed 99-loop that clashes with substrate binding in S2–S4 (Fig. 6*B*). A KLK3 substrate complex structure (Protein Data Bank code 2ZCK) suggests that the 99-loop closes like a lid on the S2–S4 subsites. In case of glyco-KLK2 the single glycan at Asn-95 could regulate the loop dynamics upon substrate binding by stabilizing the closed state (Fig. 6*B*). Moreover, the stabilizing and rigidifying effect of the glycan on the lidlike 99-loop, might be related to the open E and closed E* states of the conformational selection model, influencing the equilibrium of both states (9). These considerations can explain the turnover of small substrates, whereas the more efficient processing of protein substrates, such as pro-KLK2 or fibronectin, by glyco-KLK2 is more complex and certainly regulated by additional parameters, such as exosite binding.

**FIGURE 7.**
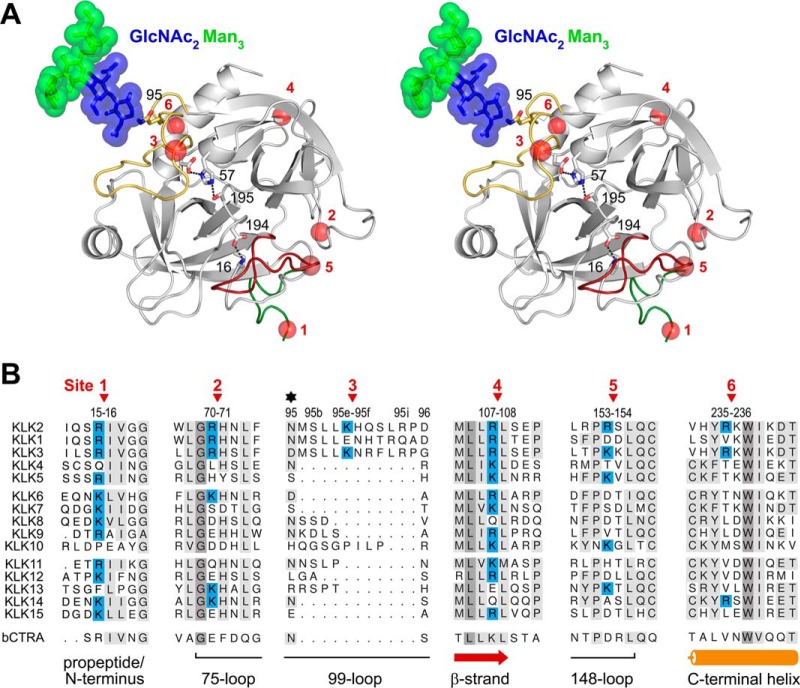
**Inactivating cleavage sites of KLK2.** *A*, *stereo representation* of a glyco-KLK2 model. The active site residues Asp-102, His-57, and Ser-195, and the stabilizing salt bridge Ile-16–Asp-194 are represented as *sticks*. The N terminus with the beginning of the propeptide (*green*), the 99-loop (*yellow*), and the 148-loop (*red*) are highlighted as specified. The six cleavage sites at Arg-15, Arg-70, Lys-95e, Arg-107, Arg-153, and Arg-226 are shown as *red spheres*. The glycan at Asn-95 that shields the 99-loop from cleavage is depicted with *sticks* and *spheres. B*, sequence alignment the six KLK2 cleavage sites, as shown in *A*, with the other 14 human tissue kallikrein family members and bovine chymotrypsin A (*bCTRA*) as a numbering reference. The *red arrows* indicate the autolysis sites on KLK2, whereas its secondary structure is depicted at the *bottom*, and the glycosylation site at Asn-95 is marked with a *star*. In contrast to KLK1 and -2, KLK3 is not glycosylated in the 99-loop but at Asn-61. Identical and conserved residues are highlighted with a *gray background*, whereby a *darker gray* indicates a higher degree of conservation. The basic P1 residues Arg and Lys in KLK2 and in the potential cleavage sites of other KLKs are highlighted with a *blue background*.

The strong enzymatic preference for P1-Arg residues of KLK2 enables the pro-form to undergo autoactivation (9, 12, 16, 21, 45). Both glyco-KLK2, which was secreted as inactive zymogen (Fig. 1*A*), and enzymatically deglycosylated KLK2 exhibit a similar autoactivation pattern (Fig. 2*A*). The difference from the quick autoactivator pro-KLK_e_ could be explained by a lower activity of glyco-KLK2 and higher stability of pro-KLK2. Given the higher activity of glyco-KLK2 as compared with KLK2_e_ toward glycosylated pro-KLK2 (Fig. 2), the lower efficiency in autoactivation of the *Leishmania* expressed proteins depends most likely on the higher stability of pro-KLK2 as substrate. Several KLKs harbor either Arg-15 or Lys-15 in the P1 position of their propeptide, making them ideal targets for KLK2 (Fig. 7), as observed for pro-KLK1, -2, and -3, in contrast to pro-KLK5, -9, -11, and -12 (9, 45, 53–55).

Five internal cleavage sites of KLK2 have been reported, including Arg-70↓His-71, Lys-95e↓His-95f, Arg-107↓Leu-108, Arg-153↓Ser-154, and Arg-235↓Lys-236, which are located on the protein surface (9, 12, 45) (Fig. 7*A*). Cleavages at Lys-95e↓His-95f in the 99-loop (9) and Arg-153↓Ser-154 in the 148-loop inactivate KLK2 (18, 45) (Fig. 3). Cleavage of Lys-95e↓His-95f (Fig. 7, site 3), located near the S2 pocket (9), generates N and C termini, which may clash with the active site or distort the catalytic triad at Asp-102. Two mutants of KLK2_e_, K95eM and K95eQ, were more resistant against autolysis in the 99-loop (9), while they were still slowly inactivated, probably due to cleavages at other sites. It is noteworthy that Lys-95e is close to Asn-95, whose glycan may shield the 99-loop and shift the preferred cleavage to Arg-153↓Ser-154 (18, 45). This inactivation site in the 148-loop (Fig. 7, site 5) might disturb the adjacent activation pocket with the N-terminal Ile-16 to Asp-194 salt bridge, which is essential for catalysis, because its disruption inactivates serine proteases (56–59). The same cleavage site with a Lys–Lys bond is present in KLK3 (60, 61). Given the similarity of the cleavage site architecture of KLK2 and -3 (45), KLK2 may inactivate KLK3 in similar manner, despite a proposed role of KLK2 as physiological activator of KLK3 (45, 62). The peptide bond between Arg-70 and His-71 of KLK2 (Fig. 7, site 2) has been shown to be targeted by FXa, which is also present in seminal plasma (12, 63). This site is located at the 75-loop, near the cleavage site Arg-153↓Ser-154 (Fig. 7, site 5), but with less solvent exposure and accessibility, which may explain why Arg-70↓His-71 cleavages were not observed for KLK1 and -3. The autocleavage sites of KLK2 between residues Arg-107 and Leu-108 (Fig. 7, site 4) and residues Arg-235 and Lys-236 are located within α-helices (12, 45, 64). The Arg-107↓Leu-108 cleavage site is highly conserved among human KLK1, -3, -6, -9, -12, and -15 (Fig. 7, site 4), whereas Arg-235↓Lys-236 is only found in KLK3 (Fig. 7, site 6).

Aside from proteolytic processing, protein glycosylation can markedly influence protein conformation, function, and regulation. Apart from a dampening effect on the movements of the flexible 99-loop, the glycan most likely induces the formation of a type I β-turn, in particular when two GlcNAc are present (65, 66), in place of the Asx turn of in KLK2_e_. The general significance of glycans for enzymatic activity and physiological regulation can be corroborated by examples, such as the trypsin-like human tissue-type plasminogen activator, which possesses three *N*-glycosylation sites, one in the protease domain. Completely aglycosylated tissue-type plasminogen activator is up to 4-fold more active against chromogenic substrates (67), whereas a higher mannose content or removal of the terminal sialic acids enhances the activity (68). Otherwise, effects of *N*-glycosylation on structure and function may be negligible in some cases as for partially deglycosylated human complement factor I (69). However, there are numerous examples for marked *N*-glycosylation effects from the protease field alone, such as deglycosylated plasma kallikrein (KLKB1) and its glyco-form, which exhibit considerably different cleavage patterns for protein substrates (70). Also, natural, inhomogeneously glycosylated KLK3 and deglycosylated KLK3 from recombinant expression are equally active, whereas recombinant glyco-KLK3 was 3 times more active against small chymotryptic substrates (71). Altered glycosylation patterns of KLK3 (31) and KLK6 (32, 72) have been related to prostate and ovarian cancer, respectively. Mass spectrometry identified up to 40 different glycan structures on KLK3 (73, 74) and 11 on KLK6 (72), whereas it is hard to determine the functional role of specific natural glycosylation sites. On the amino acid sequence level, glyco-KLK2 is identical to non-glycosylated KLK2_e_, resulting in similar biochemical properties; both pro-KLK2 forms autoactivate, both proteases process the natural substrate fibronectin in a similar manner, and both inactivate via internal cleavage. Besides these similarities, glycosylation has several distinguishing effects on KLK2: (*a*) the stability of pro-KLK2 increases without affecting autoactivation; (*b*) apparently, the active site conformation altered (Fig. 6) and concomitantly the activity toward small substrates; (*c*) the 99-loop, which is the major inactivation site in KLK2_e_, is shielded from cleavage, explaining the retarded autolysis; and (*d*) the altered enzymatic activity generates different cleavage patterns of pro-KLK2 as substrate. Analogously, this altered KLK2 activity may serve as a control mechanism for KLK3 *in vivo*. Remarkably, the percentage of clipped free KLK3 is highly related to benign prostatic hyperplasia (75). Alternative protein cleavage and glycosylation of KLK2 may correlate with the disease state, suggesting that KLK2 monitoring may increase the sensitivity and specificity of prostate cancer markers.

## Author Contributions

S. G. conducted most of the experiments, analyzed the results, and wrote major parts of the paper. W. S. prepared the KLK2e samples and measured their activity. V. M. prepared PICS samples, conceived the study, and analyzed data. P. B. did the mass spectrometry experiments and analysis. M. L. B. and O. S. performed PICS experiments and analyzed the results. J. K. conducted N-terminal sequencing and data analysis. H. B. conceived and coordinated the study and interpreted the data. P. G. conceived and coordinated the study, performed enzyme kinetic measurements, analyzed the data, and wrote parts of the paper.

## Footnotes

4 According to the STRENDA guidelines, the identity of KLK2 should be specified as tissue or glandular kallikrein (IBUMB: EC 3.4.21.35). It corresponds to isoform 1 with the UNIPROTKB sequence identifier P20151-1 (KLK2_HUMAN).

5 M. L. Biniossek, M. Niemer, K. Maksimchuk, B. Mayer, J. Fuchs, P. F. Huesgen, D. G. McCafferty, B. Turk, G. Fritz, G. Haecker, L. Mach, and O. Schilling, manuscript in preparation.

## Footnotes

3 The abbreviations used are:

KLK: kallikrein-related peptidase
KLK2_e_: *E. coli* KLK2
AMC: 7-amino-4-methylcoumarin
*p*NA: *para*-nitroaniline
PPACK: H-D-Phe-Pro-Arg chloromethyl ketone
PICS: proteomic identification of protease cleavage sites
PNGase F: peptide-*N*^4^-(*N*-acetyl-β-glucosaminyl)asparagine amidase
LEXSY: *Leishmania* expression system
Bz: benzyl.
